# Big-five personality traits and depression: chain mediation of self-efficacy and walking

**DOI:** 10.3389/fpsyt.2024.1460888

**Published:** 2024-11-29

**Authors:** Han Cai, Hongtao Song, Yating Yang, Zihe Xiao, Xianlong Zhang, Feng Jiang, Huanzhong Liu, Yi-lang Tang

**Affiliations:** ^1^ Department of Psychiatry, Chaohu Hospital of Anhui Medical University, Hefei, China; ^2^ Department of Psychogeriatrics, The Fourth People’s Hospital of Wuhu, Wuhu, China; ^3^ School of Mental Health, Bengbu Medical University, Bengbu, China; ^4^ Department of Child and Adolescent Psychology, The Second People's Hospital of Huizhou, Huizhou, China; ^5^ Department of Psychiatry, School of Mental Health and Psychological Sciences, Anhui Medical University, Hefei, China; ^6^ School of International and Public Affairs, Shanghai Jiao Tong University, Shanghai, China; ^7^ Institute of Healthy Yangtze River Delta, Shanghai Jiao Tong University, Shanghai, China; ^8^ Anhui Provincial Key Laboratory for Brain Bank Construction and Resource Utilization, Hefei, China; ^9^ Anhui Psychiatric Center, Hefei, China; ^10^ Department of Psychiatry and Behavioral Sciences, Emory University, Atlanta, GA, United States; ^11^ Atlanta Veterans Affairs Medical Center, Decatur, GA, United States

**Keywords:** personality, depression, self-efficacy, walking, mediating effect

## Abstract

**Background:**

Depression is a major global public health concern, with research indicating a correlation between personality traits and depression. This study aimed to explore the potential mediating roles of self-efficacy and walking in the relationship between personality traits and depression among Chinese residents.

**Methods:**

A cross-sectional questionnaire survey was conducted from July 10 to September 15, 2021, involving 11,031 Chinese residents across 23 provinces, 5 autonomous regions, and 4 municipalities Participants provided data on demographics, personality traits (using the Ten-Item Personality Inventory), self-efficacy (using the New General Self-Efficacy Scale), chronic disease self-management (using the Chronic Disease Self-Management Study Measures), and depression (using the Patient Health Questionnaire-9). After screening, data from 8,499 participants were analyzed. Sequential mediation models were employed, with the Big Five personality traits as predictors, depression as the outcome, and self-efficacy and walking as the mediators.

**Results:**

Extraversion, agreeableness, conscientiousness, and emotional stability were negatively correlated with depression, with self-efficacy and walking as positive mediators in these relationships. Conversely, openness was positively associated with depression, and the self-efficacy-walking chain did not mediate this relationship but rather masked the effect of openness on depression.

**Conclusions:**

Our findings suggest that self-efficacy and walking are significant mediators in the relationship between personality traits and depression, potentially mitigating the risk of depressive episodes.

## 1 Introduction

Depressive disorder, a prevalent mental health condition, impacts a substantial portion of the global population, exceeding 300 million individuals and representing around 4.4% of the world’s inhabitants (1). Depression affects individuals across all age groups (2), leading to diminished quality of life, increased medical comorbidities, and elevated mortality rates. Alarmingly, treatment rates for depressive disorders remain very low, with few individuals receiving adequate care (3).

Previous studies have explored various aspects of depression among adolescents and adults by considering a range of explanatory variables, encompassing both individual and contextual predictors (4–7). An influential theoretical framework, the Five Factor Model (8), has primarily delved into individual predictors of depression, with a particular focus on the relationship between personality traits and depressive symptoms. This model posits that personality comprises five key traits: 1) extraversion, capturing characteristics such as enthusiasm, sociability, decisiveness, activity, adventurousness, and optimism (9); 2) agreeableness, encompassing qualities like trust, altruism, straightforwardness, compliance, modesty, and empathy (9); 3) conscientiousness, reflecting attributes such as competence, fairness, organization, diligence, achievement orientation, self-discipline, prudence, and restraint (10); 4) neuroticism, which denotes a general tendency towards emotional instability and includes features such as anxiety, hostility, vulnerability, self-consciousness, impulsivity, and susceptibility (11); and 5) openness, which centers on a predisposition towards imagination, aesthetics, emotional depth, curiosity, creativity, and intellect (9).

Research by Weinstock et al. confirmed that neuroticism is positively correlated with affective disorders (12), while extroversion and conscientiousness demonstrate negative correlations with these disorders. Furthermore, the impact of social network size has been observed to moderate the associations between extraversion, agreeableness, and depression (13). Current evidence also suggests that conscientiousness, coupled with cognitive reappraisal strategies, may mitigate anxiety and depression symptoms among Chinese firefighters (14). These findings collectively underscore the intricate nature of the relationship between the big five personality traits and depression, emphasizing the need for further exploration and understanding.

Another intriguing avenue of inquiry delves into the interplay between depression and self-efficacy, a pivotal factor in the cultivation of resilience (15). Self-efficacy denotes an individual’s confidence in their ability to attain desired outcomes through their actions, influencing motivation, goal establishment, and perseverance in the face of challenges, thereby intricately tied to depression (16). Self-efficacy has been observed to be positively correlated with extraversion, conscientiousness, agreeableness, and openness, while negatively correlated with neuroticism among individuals diagnosed with cancer (17). Studies focusing on university graduate employees have indicated that heightened levels of self-efficacy predict enhanced academic performance, and hope (18), as well as adaptive and pro-social behaviors (19). Furthermore, self-efficacy has been linked inversely to depression (16) and serves a pivotal role in the application of functional skills in the daily lives of individuals with Major Depressive Disorder (MDD).

The inverse relationship between physical activity and depression is well-documented (20). Walking, a straightforward and natural form of physical exercise, proves to be well-received by older adults and those with physical limitations. The advantages of walking extend to improved cognitive well-being and a diminished risk of stress, depression, and dementia (21, 22). While self-efficacy and walking represent distinct domains, their functions are intertwined (23). Individuals with lower levels of self-efficacy exhibit reduced engagement in walking behaviors (24). Although self-efficacy is associated with, and predictive of, walking habits, these two factors demonstrate partial overlap without complete concordance (25, 26).

Drawing from the aforementioned literature, primary research has centered on unraveling the interplay between personality traits and depression (27), personality traits and self-efficacy (28), as well as personality traits and walking habits (29). Some studies have explored the connection between depression and self-efficacy (30), while others have delved into the relationship between depression and walking exercise (31). Nonetheless, there remains a dearth of studies focusing on the mediating roles of self-efficacy and walking in the nexus between personality traits and depression.

Despite the wealth of existing literature, a coherent consensus is yet to emerge regarding the complex dynamics among personality traits, self-efficacy, walking, and depression. Regarding personality traits, the majority of studies indicate their association with physical activity, with extraversion emerging as the most predictive factor for engaging in physical exercise (32). However, integrative models of personality and health indicate that these fundamental traits impact health outcomes indirectly through cognitive and/or behavioral mechanisms. One possible pathway by which personality traits influence the development of mental disorders is through their relationships with physical activity (33). Consequently, these findings prompt the hypothesis that overarching self-efficacy and overall physical activity serve as potential mediators intricately linked in a defined sequence to explicate the relationship between personality traits and depression.

In consideration of these insights and within the theoretical framework of the big-five personality traits, this study aims to scrutinize the connections encompassing personality traits and pertinent factors implicated in depression. Specifically, the inquiry delves into the extent to which the interrelations between personality traits and depression are mediated by the interplay between self-efficacy and walking behaviors within a cohort of Chinese residents. Consistent with prior research, the anticipation is to uncover that the self-efficacy-walking causal pathway stands as a viable mediator in illuminating the correlation between the examined personality traits and depression.

## 2 Methods

### 2.1 Data and participants

The data utilized in this study were derived from the Psychology and Behavior Investigation of Chinese Residents (PBICR) (34, 35), conducted from July 10 to September 15, 2021. This study used multi-stage sampling method. their began by including the provincial capitals of 23 provinces and 5 autonomous regions in China, along with 4 municipalities(Beijing, Tianjin, Shanghai, Chongqing). From non-capital cities in each province, 2-6 cities were selected using a random number table method, resulting in a total of 120 cities. Next, investigators of survey teams (comprising ≤ 10 people for each team) started open recruitment in these cities. Using data from the “2021 Seventh National Population Census”, quota sampling was applied to ensure that the urban residents sampled reflect the demographic characteristics of gender, age, and urban-rural distribution. Each city must recruit at least one investigator or survey team, with each investigator responsible for collecting 30 to 90 questionnaires and each team tasked with collecting 100 to 200.

Potential participants were approached in two primary ways, depending on the COVID-19 situation and logistical constraints in each community (36): Community Health Service Centers: In locations where in-person interaction was feasible, investigators set up recruitment stations in local health service centers or related community health stations. Recruitment posters were displayed, and both paper and electronic recruitment notices were distributed to attract participants. Individuals who were interested in participating could approach the investigators directly at these centers. One-on-One Recruitment via Electronic Means: In instances where face-to-face recruitment was not possible due to pandemic restrictions, participants were recruited through community networks using electronic means. Investigators reached out to potential participants via instant messaging platforms (e.g., WeChat), and the study information was shared through community groups. Eligible individuals were then guided through the process of providing consent and completing the questionnaire via video calls (e.g., through Tencent Meeting).

The surveyor utilized the online questionnaire platform, Wenjuanxing (https://www.wjx.cn/), to distribute questionnaires one-on-one and face-to-face to the public in their respective areas of responsibility. Survey participants responded by clicking on the provided link. Informed consent was obtained from the respondents during the survey, and the surveyor entered the questionnaire number. If a respondent had the ability to think but lacked sufficient mobility to answer the questionnaire, the surveyor would conduct one-on-one interviews and answer the questions on their behalf. Prior to data collection, participants provided informed consent detailing the study’s objectives, confidentiality, anonymity, and related rights. Ethical approval for the research was granted (JNUKY-2021-018) after review.

The study’s inclusion criteria specified individuals aged over 18 years, identifying as ethnically Chinese and holding permanent residency status in China with limited annual travel (≤1 month). Prospective participants were required to volunteer for the study, provide informed consent, demonstrate the ability to independently complete either online or paper-based questionnaire surveys, and possess a clear understanding of the questionnaire content. On the contrary, exclusion criteria comprised individuals exhibiting delirium, abnormal behavior, or involvement in concurrent research projects of similar nature, as well as those expressing a lack of willingness to collaborate.

After collecting the questionnaires, two reviewers conducted comprehensive logic checks and data screening. The criteria for screening included: 1. Questionnaires completed in less than 240 seconds; 2. Inconsistent responses, including: (a) indicating “≤18 years of age” in question 16 while selecting “husband and wife family,” “DINK family,” or “single-person family” in question 3; (b) indicating “≤18 years of age” in question 16 and selecting “married,” “divorced,” or “widowed” in question 21; (c) indicating “≤18 years of age” in question 16 and then listing “spouse,” “father-in-law,” or “mother-in-law” in question 25; (d) inconsistency in reported height, weight, or BMI; 3. Incomplete questionnaries; 4. Duplicated submissions; and 5. Uniform or excessively similar responses across all checked options.

The initial survey included 11,031 individuals. Following data screening, 1,487 participants with psychiatric disorders, dementia, physical disabilities, or severe medical conditions were excluded from the analysis. As a result, the final cohort for analytical purposes comprised 8,449 participants.

### 2.2 Measurements

#### 2.2.1 Demographic characteristics

The dataset encompassed variables such as gender, age, education level, ethnicity, place of residence, marital status, household income (monthly per capita in Yuan), presence of chronic diseases, medication use, and BMI. Age categories included individuals aged 19-35, 36-50, 51-65, and over 65 years. Residents were categorized based on rural or urban locality within their region. Marital status options included single, married, divorced, or widowed. Education levels were delineated by years of schooling completed. The variables for chronic diseases and medication usage were defined as none, one, two or more. Alcohol consumption and smoking habits were classified as never, former, or current consumers.

#### 2.2.2 Assessment of walking

The Chronic Disease Self-Management Study Measures (CDSSM) is a universal tool for self-assessment of chronic disease management, developed by Lorig in 1999 (37). CDSSM comprises three main components: self-management behaviors, self-efficacy, and health outcomes. Within the realm of self-management behaviors, exercise plays a significant role and includes activities such as stretching, walking, swimming, cycling, and the use of aerobic exercise equipment like stairmasters, rowing machines, or skiing machines. Specifically, walking is quantified by weekly time intervals ranging from 0 (never) to 4 (more than 3 hours). The translation of this scale into Chinese, conducted by Siu (38), was rigorously tested for reliability, validity, and cultural appropriateness, establishing its wide acceptance in China. The scale demonstrated a Cronbach’s alpha coefficient of 0.801, indicating good internal consistency.

#### 2.2.3 Assessment of depression symptoms

The assessment of participants’ depression symptoms was conducted using the Patient Health Questionnaire-9 (PHQ-9) (39). Each item on the questionnaire is rated on a 4-point Likert scale, ranging from 0 (never) to 3 (nearly every day). The PHQ-9 total score ranges from 0 to 27, with higher scores indicating greater severity of depression symptoms. In this study, the PHQ-9 exhibited high internal consistency, with a Cronbach’s alpha coefficient of 0.938.

#### 2.2.4 Assessment of self-efficacy

In assessing participants’ self-efficacy, the New General Self-Efficacy Scale (NGSES) was utilized (40). Each item on the scale is rated on a 5-point Likert scale, from 1 (strongly disagree) to 5 (strongly agree). The total NGSES score falls between 1 and 40, with higher scores correlating with greater self-efficacy levels. This study demonstrated a high internal consistency of the NGSES, as indicated by a Cronbach’s α coefficient of 0.944.

#### 2.2.5 Assessment of big-five personality traits

In this study, the Ten-Item Personality Inventory (TIPI) was used to evaluate the five-factor model (FFM) personality traits (41). The brief Chinese version of the TIPI (42, 43), developed by Gosling (44), consists of 10 items rated on a scale from 1 (strongly disagree) to 5 (strongly agree). Each of the Big Five-factor dimensions (E-Extraversion, A-Agreeableness, C-Conscientiousness, EI-Emotional Stability, and O-Openness) is represented by two items, with one item in each pair being reverse-coded for dimension score calculation. The reverse-scored items are 2, 4, 6, 8, and 10. Higher scores indicate higher levels of the respective traits. The Cronbach’s alpha coefficients in this study were 0.783 for extraversion, 0.770 for agreeableness, 0.791 for conscientiousness, 0.760 for emotional stability, and 0.766 for openness.

### 2.3 Statistical analysis

The data underwent processing and analysis using Microsoft Excel, SPSS 25.0, and Mplus Version 8.3 (45). Initially, Microsoft Excel was employed for data preprocessing, encompassing tasks such as data refinement and total score computation. Subsequently, SPSS 25.0 was utilized for assessing common method bias, conducting descriptive statistical analyses, and determining correlations. Descriptive statistics were used to analyze the demographic characteristics, while Pearson’s correlation analysis was performed to estimate the associations among personality traits, self-efficacy, walking, and depression. Normally distributed continuous variables were reported as the means and SDs, whereas non-normally distributed continuous variables were reported as medians and interquartile ranges (IQRs). Categorical variables were presented as frequencies and percentages. Linear correlation was used to analyze the relationship between the five personality traits, self-efficacy, walking, and depression. Finally, Mplus software was employed to construct a structural equation model and evaluate its mediating effects. We performed the bootstrapping procedure by using one independent variable, two mediators, and one dependent variable. We calculated 95% confidence intervals (CIs), based on bias-corrected bootstrap analyses with 5000 repetitions to analyze indirect effects. In the present research, we tested the proposed mediation models considering two mediators in the chain. Within this framework, the independent variables consisted of the big-five personality traits, with depression serving as the dependent variable and self-efficacy and walking as mediating factors. Additionally, gender, education, marital status, ethnicity, residence, household income, chronic illnesses, medication usage, alcohol consumption, smoking habits, age, and BMI were included as controlled variables. When performing mediation analysis, it is important to include control variables in the model statements and ensure they influece all relevant outcome variables. First, we calculated the direct effects of depression on personality traits, self-efficacy, and walking. Next, we examined the direct effects of walking on personality traits and self-efficacy, followed by the direct effects of self-efficacy on personality traits. Finally, we assessed the indirect effects of depression on personality traits. The results were considered statistically signifcant when p<0.05. An indirect effect was considered signifcant when the confidence interval did not include zero.

## 3 Results

### 3.1 Common methods bias test

Common method bias (CMB) denotes the spurious correlation between predictor and criterion variables arising from shared data sources, raters, measurement environments, project contexts, and project characteristics (46, 47). This form of spurious correlation can significantly confound research outcomes and potentially lead to erroneous conclusions, constituting a form of systematic error. CMB is prevalent in psychological and behavioral science research, particularly in studies employing questionnaire methodologies. The assessment of common method bias often involves the application of Harman’s one-factor test, whereby its presence is indicated by a single or dominant factor explaining most of the variance. In the current investigation, a factor analysis loading all items on an unrotated factor revealed that the initial principal factor accounted for merely 27.61% of the variance, well below the 50% threshold (48). Hence, it can be concluded that common method variance was not a factor in the present study.

### 3.2 Sample statistics

A cohort of 8,499 residents was the focal point of this study (**Table 1**). Among these individuals, the majority were women, comprising 56.5% of the sample, while 32.4% fell within the 36-50 age bracket. A significant proportion of the respondents resided in urban settings (73.5%). With respect to ethnicity, the vast majority were of Han descent (94.3%), and 74.8% boasted an educational attainment of high school level or above. Furthermore, 39.1% of participants reported a monthly per capita family income ranging from 3001 to 6000 yuan. Regarding health status, the majority (84.3%) reported no chronic illnesses, and a notable proportion (80.5%) reported no regular medication usage. A considerable 59.3% refrained from alcohol consumption, and 81% reported never having smoked. The mean Body Mass Index (BMI) of the cohort was recorded at 22.14 (SD 3.13).

**Table 1:** Socio-demographic characteristics and clinical features of the group (N=8,449).

Characteristics	N	%
Gender		
Men	3678	43.5
Women	4771	56.5
Age (years)		
19-35	4045	47.9
36-50	2738	32.4
51-65	1040	12.3
≥66	626	7.4
Educational level		
No formal education	2404	2.8
Elementary school	507	6.0
Middle school	1385	16.4
High school and above	6317	74.8
Marital status		
Single	2930	34.7
Married	5203	61.6
Divorced/Widowed	316	3.7
Ethnicity		
Han	7967	94.3
ethnic minories	482	5.7
Residence		
Rural	2238	26.5
Urban	6211	73.5
Household income (per capita monthly, yuan)		
≤3000	2361	27.9
3001-6000	3305	39.1
≥6001	2783	32.9
Number of chronic disease		
None	7122	84.3
1	962	11.4
≥2	365	4.3
Medications		
none	7048	83.4
1	703	8.3
≥2	698	8.2
Alcohol use		
Never	5014	59.3
Former	1014	12.0
Drink	2421	28.7
Cigarette smoking		
Never	6801	80.5
Former	591	7.0
Smoke	1057	12.5
	Mean	SD
BMI	22.14	3.13
NGSES	28.69	5.13
TIPI		
Extraversion	6.29	1.62
Agreeableness	7.06	1.47
Conscientiousness	6.87	1.61
Emotional Stability	6.27	1.49
Openness	6.47	1.55
	Median	IQR
CDSSM-walking	2	1
PHQ-9	5	2

SD, standard deviation; IQR, Interquartile Range; BMI, Body Mass Index; CDSSM, Chronic Disease Self-Management Study Measures; TIPI, Ten-Item Personality Inventory; PHQ-9, Patient Health Questionnaire-9; NGSES, New General Self-Efficacy Scale.

### 3.3 Preliminary analyses

The interplay among the big-five personality traits, self-efficacy, walking behavior, and depressive symptoms is delineated in **Table 2**. Findings derived from the correlation analysis unveiled significant relationships between depression and traits such as extroversion (r = -0.119, p < 0.001), agreeableness (r = -0.198, p < 0.001), conscientiousness (r = -0.237, p < 0.001), emotional stability (r = -0.258, p < 0.001), and openness (r = -0.018, p < 0.001). Notably, scores on the PHQ-9 questionnaire exhibited strong correlations with both self-efficacy (r = -0.239, p < 0.001) and walking habits (r = -0.136, p < 0.001) as indicated in **Table 2**.

**Table 2:** Correlation analysis of CDSSM-walking, the big five personality traits, NGSES and PHQ9 (N = 8,449).

Variable	1	2	3	4	5	6	7	8
1.CDSSM-walking	1.00							
2.Extraversion	0.078***	1.00						
3.Agreeableness	0.103***	-0.016	1.00					
4.Conscientiousness	0.149***	0.164***	0.278***	1.00				
5.Emotional Stability	0103***	0.182***	0.258***	0.177***	1.00			
6.Openness	0.087***	0.168***	0.127***	0.054***	0.081***	1.00		
7.NGSES	0.174***	0.173***	0.248***	0.340***	0.239***	0.195***	1.00	
8.PHQ-9	-0.136***	-0.119***	-0.198***	-0.237***	-0.258***	-0.018***	-0.239***	1.00

***P< 0.01. CDSSM, Chronic Disease Self-Management Study Measures; PHQ-9, Patient Health Questionnaire-9; NGSES, New General Self-Efficacy Scale

### 3.4 Multiple chain mediation analysis

**Figure 1:**
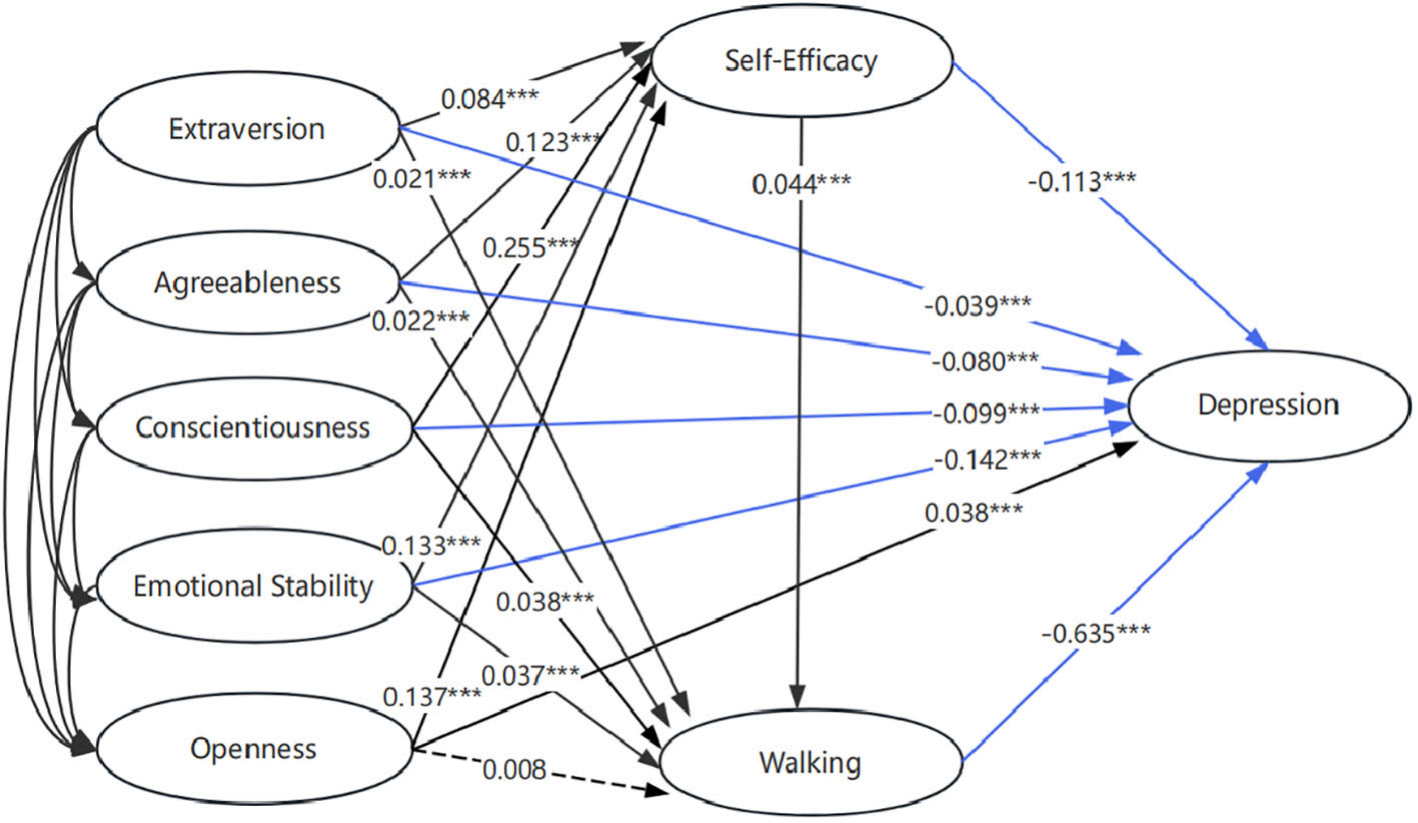
The chain mediation model of self-efficacy and walking betweeen big-five personality traits and depression symptoms. The red line indicates the positive regression coefficient. The blue line indicates the negative regression coefficient. The solid line indicates that the regression coefficient is significant (p < 0.05). The dashed line indicates that the regression coefficient is insignificant (p > 0.05). *** indicates p < 0.001.

Utilizing the Mplus software, we conducted a comprehensive examination of multi-level mediations. Path analysis unveiled the intricate associations within the model: extraversion exhibited a significant positive relationship with self-efficacy (β = 0.084, p < 0.001) and walking (β = 0.021, p < 0.001) while demonstrating a significant negative influence on depression (β = -0.039, p < 0.001). Similarly, agreeableness positively predicted self-efficacy (β = 0.123, p < 0.001) and walking (β = 0.022, p < 0.001), while negatively impacting depression (β = -0.080, p < 0.001). Moreover, conscientiousness displayed positive associations with self-efficacy (β = 0.255, p < 0.001) and walking (β = 0.038, p < 0.001), while exerting a negative effect on depression (β = -0.099, p < 0.001). Emotional stability demonstrated significant positive relationships with self-efficacy (β = 0.133, p < 0.001) and walking (β = 0.037, p < 0.001), and a negative impact on depression (β = -0.142, p < 0.001). On the contrary, openness positively influenced self-efficacy (β = 0.137, p < 0.001) and depression (β = 0.038, p < 0.001) but did not significantly predict walking (p > 0.05). Further analysis revealed that self-efficacy significantly predicted depression (β = -0.113, p < 0.001) and walking (β = 0.044, p < 0.001). Notably, walking emerged as a significant negative predictor of depressive symptoms (β = −0.635, p < 0.001) acting as a mediator (**Figure 1**). To evaluate the significance of the direct effects depicted in **Table 3**, we employed bias-corrected bootstrap tests (creating 5,000 bootstrap samples with a 95% confidence interval). The results indicated that if the 95% confidence interval of the direct path coefficient did not encompass 0, the direct path was considered significant. Analyzing total indirect effects, self-efficacy and walking were identified as partial mediators in the relationships between extraversion, agreeableness, conscientiousness, emotional stability, and depression. Notably, significant indirect effects were observed for extraversion, agreeableness, conscientiousness, and emotional stability on depression through self-efficacy, as well as for extraversion, agreeableness, conscientiousness, and emotional stability on depression through walking. The interplay of extraversion, agreeableness, conscientiousness, and emotional stability on depression through sequential pathways involving self-efficacy and walking was also supported by our findings. In the final model, the positive coefficient between openness and depression was contrasted by the negative coefficient of openness×self-efficacy×walking, revealing a masking effect rather than a mediating influence. This intricate relationship between self-efficacy, walking behavior, openness, and depression warrants further exploration in future studies.

**Table 3:** Indirect effect of big-five personality on depression via self-efficacy and walking (N = 8,449).

Paths	Standardized β (SE)	Standardized 95% CI	
		Low	High
Direct paths			
Extraversion→depression	-0.039 (0.009)	-0.022	-0.015
Agreeableness→depression	-0.080 (0.010)	-0.098	-0.064
Conscientiousness→depression	-0.099 (0.010)	-0.115	-0.082
Emotional Stability→depression	-0.142 (0.010)	-0.158	-0.126
Openness→depression	0.038 (0.009)	0.023	0.054
Indirect paths			
Extraversion→Self-efficacy→depression	-0.009 (0.002)	-0.012	-0.007
Agreeableness→Self-efficacy→depression	-0.014 (0.002)	-0.017	-0.011
Conscientiousness→Self-efficacy→depression	-0.029 (0.003)	-0.034	-0.024
Emotional Stability→Self-efficacy→depression	-0.015 (0.002)	-0.019	-0.012
Openness→Self-efficacy→depression	-0.015 (0.002)	-0.019	-0.012
Extraversion→walking→depression	-0.013 (0.004)	-0.020	-0.007
Agreeableness→walking→depression	-0.014 (0.004)	-0.020	-0.007
Conscientiousness→walking→depression	-0.024 (0.004)	-0.031	-0.018
Emotional Stability→walking→depression	-0.024 (0.004)	-0.030	-0.017
Openness→walking→depression	-0.005 (0.004)	-0.011	0.001
Extraversion→Self-efficacy→walking→depression	-0.002 (0.000)	-0.003	-0.002
Agreeableness→Self-efficacy→walking→depression	-0.003 (0.001)	-0.004	-0.002
Conscientiousness→Self-efficacy→walking→depression	-0.007 (0.001)	-0.009	-0.005
Emotional Stability→Self-efficacy→walking→depression	-0.004 (0.001)	-0.005	-0.003
Openness→Self-efficacy→walking→depression	-0.004 (0.001)	-0.005	-0.003

→ represent causal relationships between variables.

## 4 Discussion

This study represents a pioneering large-scale investigation into the interplay among big-five personality traits, self-efficacy, walking, and depressive symptoms among Chinese residents. It also explored the mediating impacts of self-efficacy and walking in these relationships.

Within the array of factors associated with depression, the impact of personality traits stands as unequivocal (49), yet a comprehensive analysis of this intricate phenomenon necessitates the consideration of other related factors (50). Employing a sequential mediation model, an exploration was undertaken to ascertain whether the direct link between personality traits and depression is modulated through the causal pathway of self-efficacy-walking.

### 4.1 Main findings

In terms of direct relationships, our models revealed a notable negative correlation between depression and extraversion, agreeableness, conscientiousness, and emotional stability.

The direct negative association observed between extraversion and depression implies that individuals exhibiting higher extraversion traits, characterized by sociability and activity, tend to possess elevated resilience and reduced susceptibility to depression (51). Intriguingly, extraversion has been linked positively to self-efficacy, a cognitive process through which individuals acquire new behaviors impacting their capacity to shape future outcomes under environmental and social influences (52). This suggests that individuals with greater extraversion may be more inclined toward active coping strategies and positive emotional cognitive patterns (8). Furthermore, activity, a facet of extraversion, demonstrates a positive correlation with walking (29), known to alleviate stress and enhance mood.

In exploring the direct negative connections between personality traits and depression, we have elucidated that agreeableness plays a protective role through its association with inclusion and life satisfaction (53), as well as its inverse correlation with dysregulated states (54). Central to agreeableness are motivations to foster positive relationships, empathetic capacities toward others’ viewpoints, and inclinations toward collaboration and goal alignment. Thus, heightened levels of agreeableness serve as a safeguard against depression. Moreover, by bolstering individuals’ self-efficacy (55) and cultivating particular aspects of exercise behavior (56), one may potentially nurture prosocial tendencies that could serve as a mediated approach to mitigating depression risk.

Conscientiousness manifests through traits of orderliness, diligence, and dutifulness, forming a well-documented relationship with depression in existing literature. Individuals low in conscientiousness often encounter frustration, potentially triggering depressive symptoms (57). Additionally, the component of competence within conscientiousness, known as generalized self-efficacy, can regulate negative emotions and promote active coping, thereby mitigating the severity of depression. Furthermore, competence shows positive associations with attentional and memory biases toward positive information, factors that are inversely linked to affective disorders (8). Enhancing general self-efficacy may enhance the health-related quality of life for individuals scoring low in conscientiousness (58). Notably, conscientiousness stands out as a significant factor in exercise adherence, with a correlation observed between walking behavior and heightened conscientious traits (59). Hence, fostering self-efficacy may play a pivotal role in bolstering the protective effects associated with these traits, even among individuals demonstrating lower levels of conscientiousness.

The current research highlights emotional stability as a pivotal shield against depression. Numerous clinical studies have consistently shown a positive correlation between neuroticism and depressive symptoms (60). One potential pathway through which neuroticism influences depression is rumination, which fosters self-centered contemplation at the expense of attentive engagement with external stimuli, potentially leading to prolonged clinical anhedonia (61). Furthermore, maladaptive cognitive strategies for regulating emotions serve as mediators in the neuroticism-depression nexus (62). It has been suggested that augmenting self-efficacy and resilience may mitigate the detrimental effects of neuroticism on mental well-being (63). Additionally, neuroticism appears to be inversely linked with a preference for intense walking and adherence to walking regimens (64). Considering these findings, self-efficacy may serve as a crucial mediator in fortifying the protective influences associated with this trait, particularly in individuals exhibiting low emotional stability.

Our study revealed an intriguing finding that openness does not exhibit a negative association with depression in this particular model. Openness, defined by an individual’s inclination to embrace novel experiences and delve into diverse ideas, values, emotions, and sensations, divergent from their established preferences, could potentially facilitate active coping strategies and encourage seeking treatment for affective disorders (65). Conversely, although there was limited evidence of a positive link between openness and depression, this association may stem from the tendency of openness to lead to heightened levels of worry and wishful thinking, potentially detracting from effective planning and contributing to feelings of melancholy. Higgins (66) suggests that people who experience a greater gap between their real and ideal selves are more likely to be disappointed, dissatisfied, or sad, and thus Khoo and Simms (67) suggest that those with a higher level of openness to fantasy experience a greater gap between their ideal and real states than those with a lower level of openness to fantasy, and are more likely to be depressed. Some scholars believe that too much fantasy is not conducive to the promotion of down-to-earth practice and action, so that people escape from real problems in the fantasy world, good fantasy will cause a huge gap between expectations and reality and suffer a blow, or make the escapist more addicted to it and difficult to face the unbearable pressure of reality; And fantasizing about bad situations over and over again can also amplify emotions and immerse people in negative emotions, which is not conducive to maintaining a positive and healthy attitude. Future investigations into the relationship between openness and affective disorders may benefit from examining mediating factors. Notably, patients demonstrating higher levels of openness displayed enhanced self-efficacy concerning chronic disease management, suggesting that the open trait could promote health-promoting behaviors, such as walking, through the bolstering effects of self-efficacy, with potential implications for reducing depressive symptoms (17).

Our study aims to forge a novel preventive framework for depression by integrating concepts of meaning in psychological and physical activity to establish a chain mediation model. Our findings illuminate the chain mediating role of self-efficacy and walking in the nexus between personality traits and depression among Chinese individuals. Specifically, the mediation effect values account for 39.1% for extraversion, 27.9% for agreeableness, 38.0% for conscientiousness, and 22.7% for emotional stability, respectively, of the total effect. This implies that personality traits can exert a direct impact on residents’ depression levels while also indirectly influencing individuals’ moods by fostering self-efficacy and engagement in walking. Notably, self-efficacy emerges as the most substantial mediator in the relationship between personality and depression in Chinese residents, highlighting the potential of personality traits to mitigate depression by enhancing self-efficacy levels. Numerous studies have underscored the role of high self-efficacy in empowering individuals to navigate and derive meaning from negative thoughts and emotions, particularly in the context of comorbid depression (68). Additionally, the mediating effect of walking on the connection between personality traits and depression, while slightly lower, remains noteworthy, underscoring the favorable impact of lifestyle choices in alleviating or deterring depression. As Mensure Aydin espoused, engagement in physical activity serves as a cornerstone for enhancing various aspects of individuals’ quality of life, encompassing physical functioning, health, pain management, vitality, social interactions, fatigue, and sleep quality (69). Moreover, existing literature attests to the efficacy of walking in ameliorating depressive symptoms, warranting its prescription as an evidence-based intervention (70). Although the trait of openness may heighten vulnerability to depression, it concurrently fosters self-efficacy and walking behavior. This interplay between “self-efficacy×walking” serves to partially or fully diminish the impact of openness, thereby mitigating the risk of depression. Consequently, governmental entities are advised to devise comprehensive intervention strategies aimed at attenuating the adverse effects of openness on depression, encompassing provisions for mental health assistance, fortification of mental resilience, and promotion of active lifestyles.

### 4.2 Theoretical implications

This study makes significant theoretical contributions to the field of public mental health. First, it establishes that personality traits among Chinese residents significantly influence levels of depression. Second, both self-efficacy and walking habits partially mediate the relationship between personality and depression. Third, these factors interact within a chain mediation model linking personality traits to depressive symptoms.

These findings suggest potential intervention strategies for individuals struggling with depression and highlight the variability of depressive experiences associated with diverse personality characteristics. As studies suggested, symptoms like sleep disturbances and suicidal ideations often correlate with specific personality traits (71). While studies affirmed the impact of personality on depression and recognized personality as a precursor to depressive symptoms (72), there has been limited research on how personality influences the mediating mechanisms of depression.

Additionally, the bidirectional relationship between self-rated health and the Big Five personality traits in adolescents underscores the predictive capacity of self-perceived health on these traits. Specifically, self-rated health emerges as a significant positive predictor for extraversion, agreeableness, openness, and conscientiousness, while exhibiting a notable negative correlation with neuroticism. Conversely, neuroticism has a significantly negative predictive relationship with self-rated health, whereas openness shows a positive correlation (73).

Although some previous studies have explored the mediating roles of self-efficacy and physical activity between personality and mental health, further exploration focused on Chinese residents is warranted. This research uniquely identifies that Chinese individuals exhibiting traits such as extraversion, agreeableness, conscientiousness, and emotional stability are likely to experience lower levels of depression. Moreover, self-efficacy and walking habits are effective mediators in the relationship between personality and depression. This work enriches the understanding of how personality traits relate to depression among Chinese residents in the context of public mental health and enhances the practical implications of mental elasticity theory and healthy lifestyle theory. At the sametime, while our results are consistent with the mediating hypothesis, they do not rule out the possibility of other explanations, such as unmeasured confounding, reverse causality, or biased sample selection.

### 4.3 Practical implications

Although many projects in our research show statistically significant mediating effects, the strength of these effects is usually limited. But it still offers several practical recommendations for enhancing mental health among Chinese residents. First, given the significant impact of personality traits on depressive emotions, actionable strategies include assessing residents’ personality traits to promote mental health awareness and implementing psychological training programs in universities that encourage proactivity and strengthen personality dimensions.

Second, recognizing that self-efficacy serves as a partial mediator between personality and depression highlights the importance of enhancing residents’ social capital. Educational institutions and related organizations can promote experiences of success, establish personalized and collaborative reward systems, leverage individual strengths, and foster positive attribution styles.

Third, our findings indicate that walking habits also partially mediate the relationship between personality and depression. Encouraging regular exercise, particularly walking, is recommended for improving mental health outcomes.

Furthermore, this study elucidates the significant chain mediation role of self-efficacy and walking in the relationship between personality and depression. Strategies aimed at enhancing self-efficacy and promoting physical activity could serve as effective interventions for alleviating depressive symptoms. By refining self-efficacy regulation techniques, individuals exhibiting lower levels of extraversion or conscientiousness can better manage their emotions and behaviors. Incorporating walking into daily routines has been shown to alleviate negative emotions and reduce depressive symptoms.

As the prevalence of psychotic disorders rises, the scarcity of healthcare resources—such as psychiatrists—poses a serious challenge in ensuring equitable access to care. The risk of psychosis emerges from a complex interaction of internal and external factors, necessitating urgent attention to artificial intelligence-assisted screening for psychosis risk (74). Such innovations can enhance the efficiency and promptness of evaluations for symptomatic individuals and those at risk, enabling timely interventions and reducing the exacerbation of symptoms.

To promote optimal mental health, governments must prioritize initiatives that foster proactive behavior among residents by bolstering self-efficacy and encouraging regular walking habits. A higher level of self-efficacy can positively influence walking behaviors, resulting in enhanced mental well-being. The chain mediation model presented in this research carries significant practical implications for developing targeted interventions and improving mental health outcomes in the population.

### 4.4 Research limitations and future directions

Nevertheless, several limitations exist in this study. First, due to the inherent limitations of the cross-sectional design, we are unable to establish causal relationships for most findings (75, 76). Longitudinal studies are recommended in the future. Second, the recruitment approach may have introduced selection bias, as participants who attended health service centers or responded electronically might have been more mobile or had greater access to technology, potentially affecting the generalizability of the findings. Third, walking is the only component of CDSSM that showed a statistically significant mediation effect, while other components did not reach significance or had minimal effect sizes. We will probe into it in future study. Lastly, there may still be some variables that are not controlled in the model analysis and need to be further explored in the future.

## 5 Conclusions

Based on data from a large, representative community sample of Chinese residents, we conclude that certain personality traits, specifically extraversion, agreeableness, conscientiousness and emotional stability of the big five personality traits are protective factors against depression, with self-efficacy and walking serving as mediators that further reduce the risk of depressive symptoms. These results underscore the importance of fostering self-efficacy and promoting physical activity, such as walking, as potential strategies for mitigating depression in populations. However, the positive association between openness and depression, and the lack of mediation by self-efficacy and walking in this relationship, suggests a more nuanced interaction that merits further investigation. These insights contribute to a deeper understanding of how personality traits influence mental health and offer practical implications for public health initiatives aimed at reducing the burden of depression.

## Data Availability

The original contributions presented in the study are included in the article/supplementary material. Further inquiries can be directed to the corresponding authors.

## References

[1] Organization WH. Depression and other common mental disorders: global health estimates. Geneva: World Health Organization (2017).

[2] Hao R Jiao J Liu X Zuo J Jin H Wu Y. The effects of big five personality traits on sub-health in a Chinese young adults: A moderated mediation model. J Affect Disord. (2024) 358:335–41. doi: 10.1016/j.jad.2024.03.142 38565337

[3] Lu J Xu X Huang Y Li T Ma C Xu G. Prevalence of depressive disorders and treatment in China: a cross-sectional epidemiological study. Lancet Psychiatry. (2021) 8:981–90. doi: 10.1016/S2215-0366(21)00251-0 34559991

[4] Zhou SC Luo D Wang XQ Zhu J Wu S Sun T. Suicidal ideation in college students having major depressive disorder: Role of childhood trauma, personality and dysfunctional attitudes. J Affect Disord. (2022) 311:311–8. doi: 10.1016/j.jad.2022.05.085 35597473

[5] Ramasubbu R McAusland L Chopra S Clark DL Bewernick BH Kiss ZHT. Personality changes with subcallosal cingulate deep brain stimulation in patients with treatment-resistant depression. J Psychiatry Neurosci. (2021) 46:E490–E9. doi: 10.1503/jpn.210028 PMC8519494 34609949

[6] Jayakody K Gallagher P Lloyd AJ Cousins DA. A quantitative analysis of the relationship between affective state and personality ratings in inpatient depression (RAPID). psychol Med. (2023) 53:3416–25. doi: 10.1017/s003329172100547x 35238291

[7] Bienvenu OJ Samuels JF Costa PT Reti IM Eaton WW Nestadt G. Anxiety and depressive disorders and the five-factor model of personality: A higher- and lower-order personality trait investigation in a community sample. Depression Anxiety. (2004) 20:92–7. doi: 10.1002/da.20026 15390211

[8] Lyon KA Elliott R Ware K Juhasz G Brown LJE. Associations between facets and aspects of big five personality and affective disorders:A systematic review and best evidence synthesis. J Affect Disord. (2021) 288:175–88. doi: 10.1016/j.jad.2021.03.061 33901698

[9] Feist GJ. A meta-analysis of personality in scientific and artistic creativity. Pers Soc Psychol Rev. (1998) 2:290–309. doi: 10.1207/s15327957pspr0204_5 15647135

[10] Goldberg LR. The structure of phenotypic personality traits. Am Psychol. (1993) 48:26–34. doi: 10.1037//0003-066x.48.1.26 8427480

[11] Goldberg LR. An alternative "description of personality": The Big-Five factor structure. J Pers Soc Psychol. (1990) 59:1216–29. doi: 10.1037/0022-3514.59.6.1216 2283588

[12] Weinstock LM Whisman MA. Neuroticism as a common feature of the depressive and anxiety disorders: A test of the revised integrative hierarchical model in a national sample. J Abnormal Psychol. (2006) 115:68–74. doi: 10.1037/0021-843x.115.1.68 16492097

[13] Harada K Sugisawa H Sugihara Y Yanagisawa S Shimmei M. Big five personality traits, social networks, and depression among older adults in Japan: A multiple mediation analysis. Int J Aging Hum Dev. (2023) 97:111–28. doi: 10.1177/00914150221109893 35733353

[14] Tao YQ Liu XP Hou WX Niu HQ Wang SJ Ma ZJ. The mediating role of emotion regulation strategies in the relationship between big five personality traits and anxiety and depression among chinese firefighters. Front Public Health. (2022) 10:901686. doi: 10.3389/fpubh.2022.901686 35719646 PMC9205204

[15] Sousa LRM Leoni PHT de Carvalho RAG Ventura CAA Silva A Reis RK. Resilience, depression and self-efficacy among Brazilian nursing professionals during the COVID-19 pandemic. Ciencia Saude Coletiva. (2023) 28:2941–50. doi: 10.1590/1413-812320232810.09852023en 37878936

[16] Milanovic M Ayukawa E Usyatynsky A Holshausen K Bowie CR. Self efficacy in depression: bridging the gap between competence and real world functioning. J Nervous Ment Disease. (2018) 206:350–5. doi: 10.1097/nmd.0000000000000804 29538054

[17] Peyser T Perry LM Mossman B Xu K Kim S Moran JB. Personality and self-efficacy for illness management in cancer. Res Sq. (2024) 7:rs.3.rs-4289523. doi: 10.21203/rs.3.rs-4289523/v1 42767631

[18] Zeinalipour H. School connectedness, academic self-efficacy, and academic performance: mediating role of hope. psychol Rep. (2022) 125:2052–68. doi: 10.1177/00332941211006926 33818192

[19] Fu WQ Pan QQ Zhang WD Zhang L. Understanding the relationship between parental psychological control and prosocial behavior in children in China: the role of self-efficacy and gender. Int J Environ Res Public Health. (2022) 19:11821. doi: 10.3390/ijerph191811821 36142092 PMC9517206

[20] Imboden C Gerber M Beck J Holsboer-Trachsler E Pühse U Hatzinger M. Aerobic exercise or stretching as add-on to inpatient treatment of depression: Similar antidepressant effects on depressive symptoms and larger effects on working memory for aerobic exercise alone. J Affect Disord. (2020) 276:866–76. doi: 10.1016/j.jad.2020.07.052 32739704

[21] Roe J Mondschein A Neale C Barnes L Boukhechba M Lopez S. The urban built environment, walking and mental health outcomes among older adults: A pilot study. Front Public Health. (2020) 8:575946. doi: 10.3389/fpubh.2020.575946 33072714 PMC7538636

[22] Sipilä S Tirkkonen A Hänninen T Laukkanen P Alen M Fielding RA. Promoting safe walking among older people: the effects of a physical and cognitive training intervention vs. physical training alone on mobility and falls among older community-dwelling men and women (the PASSWORD study): design and methods of a randomized controlled trial. BMC Geriatrics. (2018) 18:215. doi: 10.1186/s12877-018-0906-0 30219032 PMC6139154

[23] Encho H Uchida K Horibe K Nakatsuka K Ono R. Walking and perception of green space among older adults in Japan: subgroup analysis based self-efficacy. Health Promotion Int. (2023) 38:daac175. doi: 10.1093/heapro/daac175 36617292

[24] Wiencierz S Williams L. Type D personality and physical inactivity: The mediating effects of low self-efficacy. J Health Psychol. (2017) 22:1025–34. doi: 10.1177/1359105315622557 26837688

[25] Jerome GJ McAuley E. Enrollment and participation in a pilot walking programme: The role of self-efficacy. J Health Psychol. (2013) 18:236–44. doi: 10.1177/1359105311430869 22419416

[26] Lee L-L Avis M Arthur A. The role of self-efficacy in older people's decisions to initiate and maintain regular walking as exercise — Findings from a qualitative study. Prev Med. (2007) 45:62–5. doi: 10.1016/j.ypmed.2007.04.011 17561246

[27] Huprich SK. Personality-driven depression: The case for Malignant self-regard (and depressive personalities). J Clin Psychol. (2019) 75:834–45. doi: 10.1002/jclp.22760 30768792

[28] Ouni S Boujelbene Y. The mediating role of big five traits and self-efficacy on the relationship between entrepreneurship education and entrepreneurial behavior: Study of Tunisian university graduate employees. Eval Program Planning. (2023) 100:102325. doi: 10.1016/j.evalprogplan.2023.102325 37290210

[29] Kekäläinen T Terracciano A Sipilä S Kokko K. Personality traits and physical functioning: a cross-sectional multimethod facet-level analysis. Eur Rev Aging Phys Act. (2020) 17:20. doi: 10.1186/s11556-020-00251-9 33292163 PMC7685629

[30] Cherewick M Hipp E Njau P Dahl RE. Growth mindset, persistence, and self-efficacy in early adolescents: Associations with depression, anxiety, and externalising behaviours. Global Public Health. (2023) 18:2213300. doi: 10.1080/17441692.2023.2213300 37196667

[31] Lin FL Yeh ML Lai YH Lin KC Yu CJ Chang JS. Two-month breathing-based walking improves anxiety, depression, dyspnoea and quality of life in chronic obstructive pulmonary disease: A randomised controlled study. J Clin Nursing. (2019) 28:3632–40. doi: 10.1111/jocn.14960 31192478

[32] de Bruijn G-J Kremers SPJ van Mechelen W Brug J. Is personality related to fruit and vegetable intake and physical activity in adolescents? Health Educ Res. (2005) 20:635–44. doi: 10.1093/her/cyh025 15781444

[33] McDowell CP Wilson KE Monroe DC McCrory C Kenny RA Herring MP. Physical activity partially mediates associations between "Big" personality traits and incident generalized anxiety disorder: Findings from the irish longitudinal study on ageing. J Affect Disord. (2020) 1:46–52. doi: 10.1016/j.jad.2020.07.124 32795714

[34] Yibo WU Fan S Liu D Sun X. Psychological and Behavior Investigation of Chinese Residents: concepts, practices, and prospects. Chin Gen Pract J. (2024). doi: 10.1016/j.cgpj.2024.07.006

[35] Liu D Fan S Huang X Gu W Yin Y Zhang Z. Study protocol: A national cross-sectional study on psychology and behavior investigation of Chinese residents in 2023, PBICR. medRxiv. (2024). doi: 10.1101/2024.03.28.24305038 PMC11671216 39735279

[36] Wang YJ Kaierdebieke A Fan SY Zhang RF Huang MJ Li H. Study protocol:A cross-sectional study on psychology and behavior investigation of Chinese residents,PBICR. Psychosom Med Res. (2022) 4:19. doi: 10.53388/202219

[37] Lorig K. Outcome measures for health education and other health care interventions. London: Sage Publications (1996).

[38] Siu AMH Chan CCH Poon PKK Chui DYY Chan SCC. Evaluation of the chronic disease self-management program in a Chinese population. Patient Educ Counseling. (2007) 65:42–50. doi: 10.1016/j.pec.2006.04.013 PMC7135159 16872789

[39] Kroenke K Spitzer RL Williams JB. The PHQ-9: validity of a brief depression severity measure. J Gen Intern Med. (2001) 16:606–13. doi: 10.1046/j.1525-1497.2001.016009606.x PMC1495268 11556941

[40] Tinaz S Elfil M Kamel S Aravala SS Louis ED Sinha R. Goal-directed behavior in individuals with mild Parkinson's disease: Role of self-efficacy and self-regulation. Clin Park Relat Disord. (2020) 3:100051. doi: 10.1016/j.prdoa.2020.100051 34113841 PMC8189568

[41] Boyle GJ. The SAGE Handbook of Personality Theory and Assessment[J]. SAGE PUBN INC (2008). doi: 10.4135/9781849200462

[42] Nasti C Intra FS Palmiero M Brighi A. The relationship between personality and bullying among primary school children: the mediation role of trait emotion intelligence and empathy. Int J Clin Health Psychol. (2023) 23:100359. doi: 10.1016/j.ijchp.2022.100359 36467264 PMC9709235

[43] Shi Z Li SP Chen G. Assessing the psychometric properties of the chinese version of ten-item personality inventory (TIPI) among medical college students. Psychol Res Behav Management. (2022) 15:1247–58. doi: 10.2147/prbm.S357913 PMC9121988 35603350

[44] Gosling SD Rentfrow PJ Swann WB. A very brief measure of the Big-Five personality domains. J Res Personality. (2003) 37:504–28. doi: 10.1016/s0092-6566(03)00046-1

[45] Muthen LK Muthén BO. Mplus user’s guide (7th Ed.). Los Angeles: Muthén & Muthén (2012).

[46] Podsakoff PM Todor WD. Relationships between leader reward and punishment behavior and group processes and productivity. J Management. (1985) 11:55–73. doi: 10.1177/014920638501100106

[47] Podsakoff PM Organ DW. Self-reports in organizational research - problems and prospects. J Management. (1986) 12:531–44. doi: 10.1177/014920638601200408

[48] Podsakoff PM MacKenzie SB Lee JY Podsakoff NP. Common method biases in behavioral research: A critical review of the literature and recommended remedies. J Appl Psychol. (2003) 88:879–903. doi: 10.1037/0021-9010.88.5.879 14516251

[49] Kotov R Gamez W Schmidt F Watson D. Linking "Big" Personality traits to anxiety, depressive, and substance use disorders: A meta-analysis. psychol Bulletin. (2010) 136:768–821. doi: 10.1037/a0020327 20804236

[50] Klein DN Kotov R Bufferd SJ. Personality and depression: explanatory models and review of the evidence. Annu Rev Clin Psychol. (2011) 7:269–95. doi: 10.1146/annurev-clinpsy-032210-104540 PMC3518491 21166535

[51] Shen ST Chen ZH Qin XM Zhang MJ Dai Q. Remote and adjacent psychological predictors of early-adulthood resilience: Role of early-life trauma, extraversion, life-events, depression, and social-support. PLoS One. (2021) 16:e0251859. doi: 10.1371/journal.pone.0251859 34166367 PMC8224918

[52] Farley H. Promoting self-efficacy in patients with chronic disease beyond traditional education: A literature review. Nurs Open. (2020) 7:30–41. doi: 10.1002/nop2.382 31871689 PMC6917929

[53] Connolly FF Sev IJ. Agreeableness, extraversion and life satisfaction: Investigating the mediating roles of social inclusion and status. Scandinavian J Psychol. (2021) 62:752–62. doi: 10.1111/sjop.12755 34155642

[54] Yang Z Li ALS Roske C Alexander N Gabbay V. Personality traits as predictors of depression across the lifespan. J Affect Disord. (2024) 356:274–83. doi: 10.1016/j.jad.2024.03.073 PMC13292815 38537757

[55] Caprara GV Alessandri G Di Giunta L Panerai L Eisenberg N. The Contribution of agreeableness and self-efficacy beliefs to prosociality. Eur J Personality. (2010) 24:36–55. doi: 10.1002/per.739 PMC2893740 20592954

[56] Box AG Feito Y Brown C Petruzzello SJ. Individual differences influence exercise behavior: how personality, motivation, and behavioral regulation vary among exercise mode preferences. Heliyon. (2019) 5:e01459. doi: 10.1016/j.heliyon.2019.e01459 31065599 PMC6496506

[57] Dirzyte A Antanaitis F Patapas A. Law enforcement officers' Ability to recognize emotions: the role of personality traits and basic needs' Satisfaction. Behav Sci. (2022) 12:351. doi: 10.3390/bs12100351 36285920 PMC9598174

[58] Axelsson M Lötvall J Cliffordson C Lundgren J Brink E. Self-efficacy and adherence as mediating factors between personality traits and health-related quality of life. Qual Life Res. (2013) 22:567–75. doi: 10.1007/s11136-012-0181-z 22544414

[59] Tolea MI Costa PT Terracciano A Ferrucci L Faulkner K Coday MC. Associations of openness and conscientiousness with walking speed decline: findings from the health, aging, and body composition study. Journals Gerontology Ser B-Psychological Sci Soc Sci. (2012) 67:705–11. doi: 10.1093/geronb/gbs030 PMC3478724 22451484

[60] Oancea R Timar B Papava I Cristina BA Ilie AC Dehelean L. Influence of depression and self-esteem on oral health-related quality of life in students. J Int Med Res. (2020) 48:300060520902615. doi: 10.1177/0300060520902615 32054371 PMC7111024

[61] Liao A Walker R Carmody TJ Cooper C Shaw MA Grannemann BD. Anxiety and anhedonia in depression: Associations with neuroticism and cognitive control. J Affect Disord. (2019) 245:1070–8. doi: 10.1016/j.jad.2018.11.072 PMC9667857 30699849

[62] Andrés ML de Minzi MCR Castañeiras C Canet-Juric L Rodríguez-Carvajal R. Neuroticism and depression in children: the role of cognitive emotion regulation strategies. J Genet Psychol. (2016) 177:55–71. doi: 10.1080/00221325.2016.1148659 27010452

[63] Mei X Wang H Wang X Wu X Wu J Ye Z. Associations among neuroticism, self-efficacy, resilience and psychological distress in freshman nursing students: a cross-sectional study in China. BMJ Open. (2022) 12:e059704. doi: 10.1136/bmjopen-2021-059704 PMC9196198 35697443

[64] Kekäläinen T Terracciano A Tirkkonen A Savikangas T Hänninen T Neely AS. Does personality moderate the efficacy of physical and cognitive training interventions? A 12-month randomized controlled trial in older adults. Pers Individ Dif. (2023) 202:111957. doi: 10.1016/j.paid.2022.111957 36776733 PMC9912828

[65] Abu Raya M Ogunyemi AO Broder J Carstensen VR Illanes-Manrique M Rankin KP. The neurobiology of openness as a personality trait. Front Neurol. (2023) 14:1235345. doi: 10.3389/fneur.2023.1235345 37645602 PMC10461810

[66] Higgins ET. Self-discrepancy: a theory relating self and affect. Psychol Rev. (1987) 94:319–40. doi: 10.1057/s41599-024-02699-x 3615707

[67] Khoo S Simms LJ. Links between depression and openness and its facets. Personal Ment Health. (2018) 12:203–15. doi: 10.5498/wjp.v12.i10.1287 29611346

[68] Adams SW Bowler RM Russell K Brackbill RM Li J Cone JE. PTSD and comorbid depression: Social support and self-efficacy in World Trade Center tower survivors 14-15 years after 9/11. Psychol Trauma. (2019) 11:156–64. doi: 10.1037/tra0000404 PMC6345605 30211599

[69] Aydin M Kose E Odabas I Meric Bingul B Demirci D Aydin Z. The effect of exercise on life quality and depression levels of breast cancer patients. Asian Pac J Cancer Prev. (2021) 22:725–32. doi: 10.31557/apjcp.2021.22.3.725 PMC8286684 33773535

[70] Pentland V Spilsbury S Biswas A Mottola MF Paplinskie S Mitchell MS. Does walking reduce postpartum depressive symptoms? A systematic review and meta-analysis of randomized controlled trials. J Womens Health. (2022) 31:555–63. doi: 10.1089/jwh.2021.0296 34704837

[71] Vittengl JR Clark LA Thase ME Jarrett RB. Stability and change in relations between personality traits and the interpersonal problems circumplex during cognitive therapy for recurrent depression. Assessment. (2022) 29:1158–71. doi: 10.1177/10731911211005183 33794674

[72] Valerio MP Blasco B Tagni F Szmulewicz AG Martino DJ. Personality disturbances in melancholic and nonmelancholic unipolar major depression A systematic review and meta-analysis. J Nervous Ment Disease. (2020) 208:810–7. doi: 10.1097/nmd.0000000000001212 33002936

[73] Cao X Ji S. Bidirectional relationship between self-rated health and the big five personality traits among Chinese adolescents: a two-wave cross-lagged study. Humanities Soc Sci Commun. (2024). doi: 10.1057/s41599-024-02699-x

[74] Cao XJ Liu XQ. Artificial intelligence-assisted psychosis risk screening in adolescents: Practices and challenges. World J Psychiatry. (2022) 12:1287–97. doi: 10.5498/wjp.v12.i10.1287 PMC9641379 36389087

[75] Vanderweele TJ. Mediation analysis: A practitioner's guide. Annu Rev. (2016) 37:17–32. doi: 10.1146/ANNUREV-PUBLHEALTH-032315-021402 26653405

[76] Maxwell SE Cole DA. Bias in cross-sectional analyses of longitudinal mediation. Psychol Methods. (2007) 12:23–44. doi: 10.1037/1082-989X.12.1.23 17402810

